# Sexual forms obtained in a continuous in vitro cultured Colombian strain of *Plasmodium falciparum* (FCB2)

**DOI:** 10.1186/s12936-020-3142-y

**Published:** 2020-02-03

**Authors:** Monica Ararat-Sarria, Cesar Camilo Prado, Milena Camargo, Laura Tatiana Ospina, Paola Andrea Camargo, Hernando Curtidor, Manuel Alfonso Patarroyo

**Affiliations:** 1 Receptor-Ligand Department, Fundación Instituto de Inmunología de Colombia (FIDIC), Bogotá, Colombia; 2 PhD Programme in Biomedical and Biological Sciences, Universidad del Rosario, Bogotá, Colombia; 3 Molecular Biology and Immunology Department, Fundación Instituto de Inmunología de Colombia (FIDIC), Bogotá, Colombia; 4 Animal Science Faculty, Universidad de Ciencias Aplicadas y Ambientales (U.D.C.A), Bogotá, Colombia; 5 School of Medicine and Health Sciences, Universidad del Rosario, Bogotá, Colombia

**Keywords:** Malaria, *Plasmodium falciparum*, FCB2, Sexual differentiation, Mosquito infectivity, Gametocyte, Oocyst

## Abstract

**Background:**

The epidemiological control of malaria has been hampered by the appearance of parasite resistance to anti-malarial drugs and by the resistance of mosquito vectors to control measures. This has also been associated with weak transmission control, mostly due to poor control of asymptomatic patients associated with host-vector transmission. This highlights the importance of studying the parasite’s sexual forms (gametocytes) which are involved in this phase of the parasite’s life-cycle. Some African and Asian strains of *Plasmodium falciparum* have been fully characterized regarding sexual forms’ production; however, few Latin-American strains have been so characterized. This study was aimed at characterizing the Colombian FCB2 strain as a gametocyte producer able to infect mosquitoes.

**Methods:**

Gametocyte production was induced in in vitro cultured *P. falciparum* FCB2 and 3D7 strains. *Pfap2g* and *Pfs25* gene expression was detected in FCB2 strain gametocyte culture by RT-PCR. Comparative analysis of gametocytes obtained from both strains was made (counts and morphological changes). In vitro zygote formation from FCB2 gametocytes was induced by incubating a gametocyte culture sample at 27 °C for 20 min. A controlled *Anopheles albimanus* infection was made using an artificial feed system with cultured FCB2 gametocytes (14–15 days old). Mosquito midgut dissection was then carried out for analyzing oocysts.

**Results:**

The FCB2 strain expressed *Pfap2g, Pfs16, Pfg27*/*25* and *Pfs25* sexual differentiation-related genes after in vitro sexual differentiation induction, producing gametocytes that conserved the expected morphological features. The amount of FCB2 gametocytes produced was similar to that from the 3D7 strain. FCB2 gametocytes were differentiated into zygotes and ookinetes after an in vitro low-temperature stimulus and infected *An. albimanus* mosquitoes, developing to oocyst stage.

**Conclusions:**

Even with the history of long-term FCB2 strain in vitro culture maintenance, it has retained its sexual differentiation ability. The gametocytes produced here preserved these parasite forms’ usual characteristics and *An. albimanus* infection capability, thus enabling its use as a tool for studying sexual form biology, *An. albimanus* infection comparative analysis and anti-malarial drug and vaccine development.

## Background

Almost half of the world’s population is at risk of malaria infection caused by the intracellular parasite *Plasmodium.* The World Health Organization (WHO) estimated 219 million cases for 2017, representing a 2 million case increase compared to 2016 [1]. Two hosts are involved in the parasite’s biological cycle: the mosquito vector (where parasite sexual differentiation occurs) and the human host (where asexual replication prevails) [2]. The parasite’s sexual phase (related to human-vector transmission) starts just after the female *Anopheles* mosquito’s uptake of infected erythrocytes having parasite sexual forms (gametocytes) after biting an infected human. Sexual form fusion (macrogamete and microgamete) occurs inside the mosquito midgut; gametes then mate to form zygotes, which transform into ookinetes and embed themselves within insect midgut epithelial cells [3, 4]. Physiological conditions occur simultaneously during the ookinete stage thereby facilitating parasite mobility and oocyst formation and maturation; ookinetes differentiate into oocysts and release large amounts of sporozoites into the haemocoel upon maturation which are responsible for mosquito vector-human transmission [3, 5].

Gametocyte blockade is thus considered a good target for vaccine development because it is intended to stall the disease at human-vector transmission level [6, 7]. Small quantities of gametocytes circulate in infected humans’ bloodstreams, being exposed to the host immune system; ookinetes could come into contact with the mosquito’s immune system that includes a complement-like immune response [8–10]. The host’s immune response gradually controls *Plasmodium* dispersal by reducing the amount of circulating gametocytes and limiting parasite development inside the mosquito vector, making this *Plasmodium* sexual stage an important biological bottleneck [11, 12]. However, even with such biological control, natural response against the parasite leaves asymptomatic human parasite carriers (i.e. an important cause of transmission) [7, 8]. Parasite culture and the biological study of sexual-stage parasite antigens are important for identifying and selecting effective vaccine candidates to stop this never-ending infection cycle [13].

It is known that infection susceptibility and disease transmission through *Anopheles* is determined by the genetic characteristics of the mosquito and the parasite [4]. Mosquito immune pressure generates genetic changes in *P. falciparum*, thereby enabling its gradual adaptation to mosquitoes from different geographical regions [11]. Parasite-vector interaction has been extensively followed up in African mosquitoes because of the ease of parasite isolate availability and the ease of establishing mosquito colonies to facilitate studying the mosquito’s immune response [14–17]. Comparative analysis of parasite compatibility with mosquitoes from Africa and Southeast Asia has found parasite adaptive mechanisms regarding each *Anopheles* species; a similar approach has been followed with the Latin-American *P. falciparum* strain 7G8 and *Anopheles albimanus* [15]. Even so, knowledge is still lacking about the interaction of other Latin-American parasite strains with New World mosquitoes [18]. This has mostly been due to difficulties in establishing *Anopheles* colonies (from the New World, especially regarding epidemiologically important mosquito strains) and also the lack of parasite strains from this geographical area having characterized sexual differentiation capability [18–21].

*Plasmodium falciparum* strains can be stimulated in vitro for gametocyte production; these sexual forms could be used in antibody invasion inhibition studies and testing candidate antigens for vaccine development [22]. Some *P. falciparum* strains (such as NF54) continuously cultured in vitro keep their sexual differentiation capability; however, many lose it because of spontaneous genetic mutations of sexual differentiation-related transcription factors, like the apetala 2-gametes (*ap2*-*g*) transcription factor [23].

A group of Colombian *P. falciparum* isolates were adapted to continuous in vitro culture more than 30 years ago; the *falciparum* Colombia Bogotá 2 (FCB2) strain (an in vitro culture-adapted isolate from Colombia’s Eastern Plains) from that group was described as having sexual differentiation capability [24]. This strain has been used for antigen analysis when developing an anti-malarial vaccine and in studies of the human immune response against the parasite [25–27]. This strain has been maintained in in vitro continuous culture since then but it was not known if it conserved its sexual differentiation ability or whether sexual forms could evolve to mature forms and infect local *Anopheles* species [24].

The purpose of this study was thus to induce Colombian FCB2 strain gametocyte production and prove its infective ability by controlled female *Anopheles* mosquito infection using an artificial feeding system involving parasitized erythrocytes. These FCB2 strain sexual forms (having mosquito infective capability) could thus be used in comparative studies with other *P. falciparum* strains for evaluating antibodies produced against antigens, representing promising candidates for blocking malarial transmission [28, 29]. This information increases knowledge about this specific Colombian parasite strain and provides another tool for developing anti-malarial drugs and vaccine candidates tackling parasite transmission.

## Methods

### *Plasmodium falciparum* in vitro culture

3D7 (BEI Resources Repository, NIAID, NIH: *Plasmodium falciparum*, Strain 3D7, MRA-102, contributed by Daniel J. Carucci) and FCB2 parasite strains were cultured with O+ human erythrocytes in RPMI 1640 culture media, supplemented with human inactivated serum, in a 90% N_2_, 5% O_2_, 5% CO_2_ atmosphere [30]. The maintenance protocol of asexual and sexual forms of Delves et al. was followed [22]. Briefly, asexual parasite form culture was checked every 48 h, maintaining 0.5% culture parasitaemia in 4% haematocrit until gametocytes became mature (gametocyte stage V) after 12 to 15 days culture involving daily medium replacement, without adding fresh erythrocytes. The gametocytes were then tested regarding their sexual differentiation capability in vitro, using 100 µL gametocyte culture (stage V) at 27 °C for 20 min [31]. Samples were then spun at 2500 rpm for 3 min, pellets were analysed by Giemsa staining and gametes were visualized at 40× using a Primo Star Carl Zeiss microscope [32].

An exflagellation test was made on gametocyte culture; 50 µL mature gametocyte culture mixed with human serum was kept at room temperature for 10 min. The cells were visualized in a Neubauer chamber at 40x using a Primo Star Carl Zeiss microscope. Exflagellation centres were counted and exflagellation percentage calculated using the following equations [22]:

$$Culture\;exflagellation\;per\;mL = mean\;of\;exflagellation\;on\;4\;grids \,\times\, 2 \left( {dilution\;factor} \right) \,\times\, 10^{4}$$

$$Erythrocytes\;per\;mL = mean\;erythrocytes\;in\;16\;small\;squares \,\times\, 100 \,\times\, 2 \left( {dilution\;factor} \right) \,\times\, 10^{4}$$

$$Percentage\;exflagellating\;cells = \frac{culture\;exflagellation\;per\;mL}{erythrocytes\;per\;mL} \times 100$$

### PCR and DNA sequencing

*Plasmodium falciparum* FCB2 strain genomic DNA was extracted from parasite in vitro culture using an UltraClean BloodSpin DNA Isolation Kit (MO BIO) and stored at − 70 °C until use. The *PfRh1* and *Pfmsp2* genes were amplified using previously reported primers for *PfRh1* [33] (*Pfrh1*F-AATACACATAATAAGAAGAACC, *Pfrh1*R-TAGTGAATGTTCGTTATCTTG) and the following primers for *Pfmsp2* (*Pfmsp2*F-AAAACATTGTCTATTATAAATTTC, *Pfmsp2*R-TGCATCATTAGTAGTTGTGG). Phusion Hot Start II High-Fidelity PCR Master Mix (ThermoFisher) in a BioRad T100 thermal cycler was used for PCR amplification. A Wizard SV Gel and PCR Clean-Up System (Promega) was used for purifying PCR products which were then sequenced using the Sanger method (Macrogen).

SnapGene software (from GSL Biotech; available at snapgene.com) was used for manually editing the DNA sequences and ClustalW for aligning them [34]. EMBL-EBI MUSCLE software was used for multiple sequence alignment against reported *Pfrh1* and *Pfmsp2* sequences from other *P. falciparum* strains (3D7, NF54, HB3, FVO, CAMP/Malaysia, Senegal, D10, IT and 7G8) and visualized using MView software.

### RNA extraction and cDNA synthesis

In vitro gametocytes (from parasite culture) were used for RNA extraction; 0.2% saponin was used in gametocyte-parasitized erythrocyte soft lysis. TRIzol LS (Invitrogen)-chloroform treatment was then used for RNA extraction, following the manufacturer’s recommendations. A SuperScript III Reverse Transcriptase kit (Thermo Fischer Scientific) was used for cDNA synthesis, following the manufacturer’s recommendations. All samples’ concentrations were measured by spectrophotometry (at 260 nm) and stored at − 70 °C until use.

### RT-PCR

Primers were designed for analysing *Pfs25* (PF3D7_1031000), *Pfg27/25* (PF3D7_1302100)*, Pfs16* (PF3D7_0406200) and *Pfap2g* (PF3D7_1222600) gene transcription, using the 3D7 sequence as reference. BLAST was used for aligning these genome sequences with other *P. falciparum* strains; Primer3web software (http://www.primer3plus.com/primer3web/primer3web_input.htm) was used for designing the primers, using conserved regions. A pair of primers was designed from each gene (*Pfs25*F-CCATGTGGAGATTTTTCCAAATGTA, *Pfs25*R-CATTTACCGTTACCACAAGTTACATTC; *Pfg27/25*F-TGACAATGTTATCTTGGACACGT, *Pfg27/25*R-CCCCTCTCTCACCTCGTATT; *Pfs16*F-CCCCTCTCTCACCTCGTATT, *Pfs16*R-CCCCTCTCTCACCTCGTATT; *Pfap2g*F-CGAATGGGAAGAGAGCATGC, *Pfap2g*R-TCGCTTTCTTGTCCATGCAA). The NCBI Primer-BLAST tool (https://www.ncbi.nlm.nih.gov/tools/primer-blast/) was used for testing primer specificity. Go Taq DNA polymerase (Promega) was used for RT-PCR which was carried out on a Bio-Rad T100 thermal cycler. All PCR amplifications were resolved on 1.5% agarose gel stained with SYBR safe.

### *Anopheles albimanus* and *Anopheles stephensi* infection and dissection

The *An. albimanus* Buenaventura strain (originated from Colombia, kindly provided by the Instituto Nacional de Salud de Colombia) and *Anopheles stephensi* STE2 strain (originated from India, kindly provided by Doctors Ana Catarina Alves, Henrique Silveira and João Pinto, from the Instituto de Higiene e Medicina Tropical, Universidade Nova de Lisboa, Portugal) mosquitoes were bred at 26 ± 2 °C temperature, 60–80% relative humidity and 12 h light/dark cycles. Adults were fed with 10% sugar solution. Two to three days after eggs hatched, a maximum of 250 larvae were placed in plastic recipients and fed daily with powdered sterilized cat food [16]. Three- to six-day-old emerged female mosquitoes were used for infection assays; this involved collecting 100–150 individuals in plastic recipients covered with fine pore black netting [35]. Gametocyte culture (14–15 days old) for mosquito feeding was pelleted and diluted at 40% haematocrit with human O+ erythrocytes, supplemented with inactivated human plasma [36]. Females were then fed for 20 to 30 min on pig membranes, using an artificial feed system. After being fed with gametocytes, mosquitoes were kept for 12 to 15 days in the aforementioned conditions [35, 36].

Parasite forms in the mosquitoes’ midgut were followed-up by dissection during different parasite growth stages. A standard 0.5% mercurochrome midgut stain was used for parasite form count. Each individual’s infection rate (percentage of mosquitoes infected in the midgut) and infection intensity (the median of oocysts in mosquito midgut) was recorded and compared to experimental groups (FCB2) [5]. In vivo gametocyte infectivity was determined by analysing oocysts’ phenotypical characteristics in mosquito midgut [17].

### Statistical analysis

STATA 14 software was used for all statistical analysis (0.05 significance level). Data was reported as percentages. A Chi^2^ test was used for establishing statistical differences between group percentages; the quantitative variables in this study were described with their respective medians and interquartile ranges (IQR). A Mann–Whitney *U* test was used for comparing median values.

## Results

### *Plasmodium falciparum* FCB2 strain molecular characterization

The *P. falciparum* FCB2 strain has been maintained for a long time under in vitro culture. *Pfrh1* [33], and *Pfmsp2* were sequenced and aligned against 3D7, NF54, FVO, HB3, Camp/Malaysia, Senegal, D10, 7G8 and IT *P. falciparum* strains for determining that the FCB2 strain was not contaminated with other *P. falciparum* strains. Some nucleotide changes are seen for *Pfrh1* (3086A>T SNP in FVO, Camp/Malaysia and Senegal strains sequences 3064T>C SNP in 3D7, NF54, HB3 and 3921A>G SNP in Camp/Malaysia and Senegal strains), as well as multiple changes in the *Pfmsp2* sequence (Additional file 1).

### *Plasmodium falciparum* FCB2 strain genetic gametocyte production capability

**Fig. 1:**
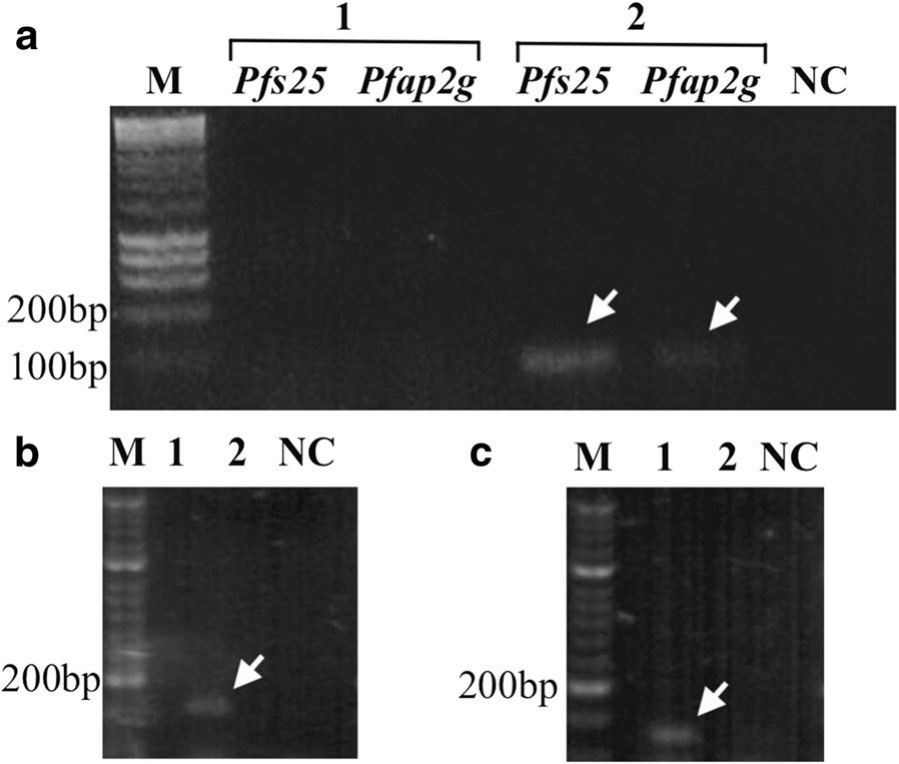
*Pfs25* and *Pfap2g* expression. RT-PCR was used for analysing *Pfs25, Pfs16, Pfg27/25* and *Pfap2g* gene expression in the in vitro cultured *P. falciparum* FCB2 strain induced for sexual differentiation. **a** FCB2 *Pfap2g* and *Pfs25* amplification; **b** FCB2 *Pfg27/25* amplification. **c** FCB2 *Pfs16* amplification. 1: Asexual parasite in vitro culture, 2: Gametocyte in vitro culture, M: molecular weight marker, NC: negative control

It is known that continuous in vitro *Plasmodium* strain culture tends to delete genes which are important for sexual differentiation, thereby decreasing or impairing parasite gametocyte production capability [23, 37, 38]. The most important genetic change associated with the loss of the parasite sexual differentiation capability is related to the deletion or absence of *Pfap2g* gene expression [37]. The *P. falciparum* FCB2 strain has been kept in continuous in vitro culture for nearly 30 years, probably losing sexual differentiation capability associated with a loss of *Pfap2g* expression. *Pfap2g* gene expression was verified by RT-PCR; *Pfap2g* gene expression was found in these parasites after having induced parasite differentiation to sexual forms in in vitro culture (Fig. 1); no *Pfap2g* expression was seen in non-induced culture (Fig. 1). RT-PCR was used to verify the FCB2 strain’s genetic sexual differentiation capability; it corroborated *Pfs25, Pfs16* and *Pfg27/25* gene transcription [39–42] by RT-PCR, amplifying the specific band (Fig. 1).

### *Plasmodium falciparum* FCB2 strain gametocyte production

**Fig. 2:**
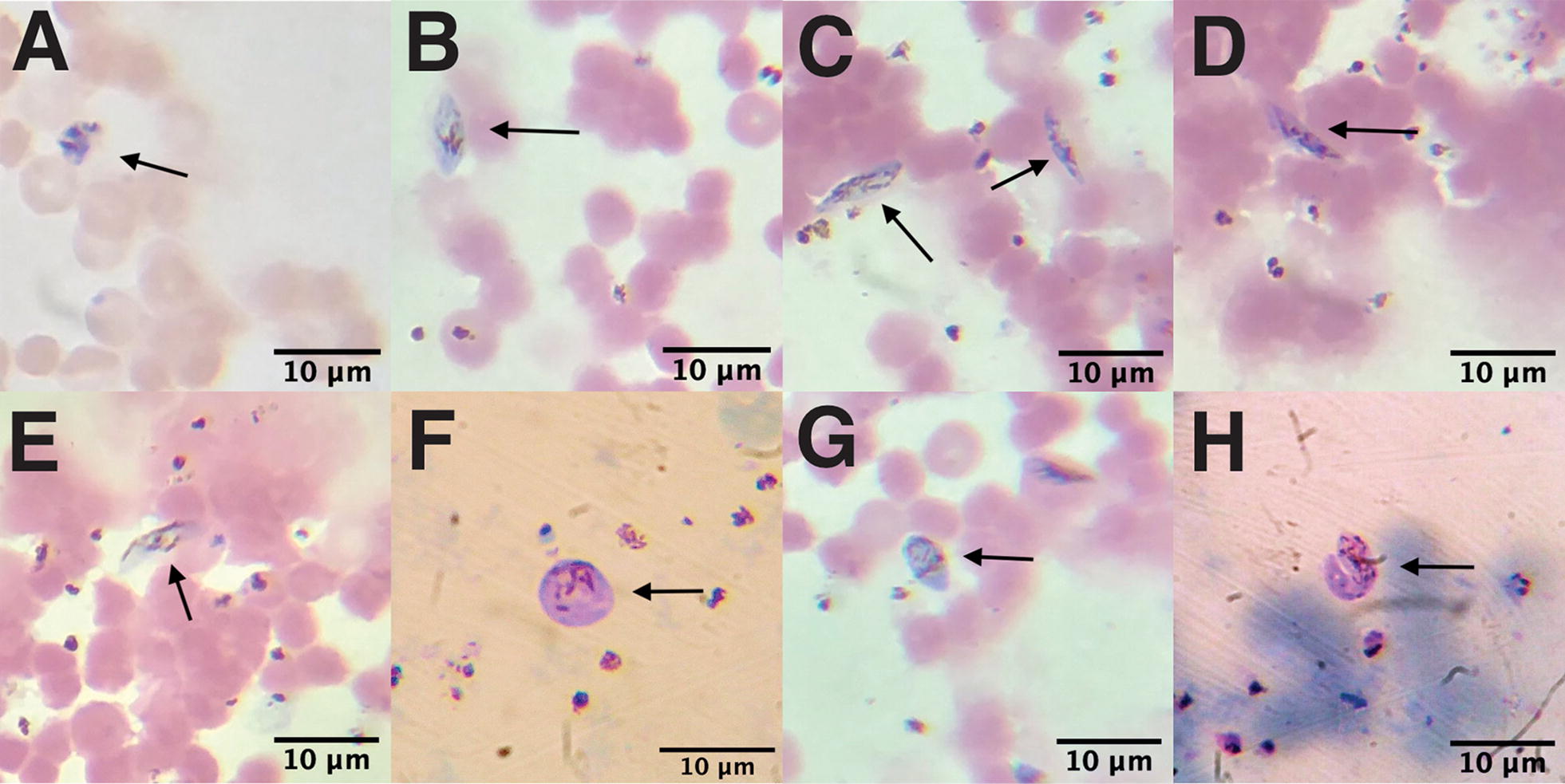
Morphological characteristics of gametocytes produced by the *Plasmodium falciparum* FCB2 strain. FCB2 strain gametocytes obtained from parasite culture at 0.3% parasitaemia were analysed by Giemsa staining. Gametocytes were visualized at 100X on a Primo Star Carl Zeiss microscope and 3 biological replicates were performed, counting minimum 3 fields per replica. Black arrows indicate parasite forms. **A** FCB2 gametocyte stage I–II; **B** FCB2 gametocyte stage II–III; **C**–**E** FCB2 gametocyte stage IV–V; **F**, **G** FCB2 form similar to zygote stage; **H** FCB2 form suggesting ookinete stage

**Fig. 3:**
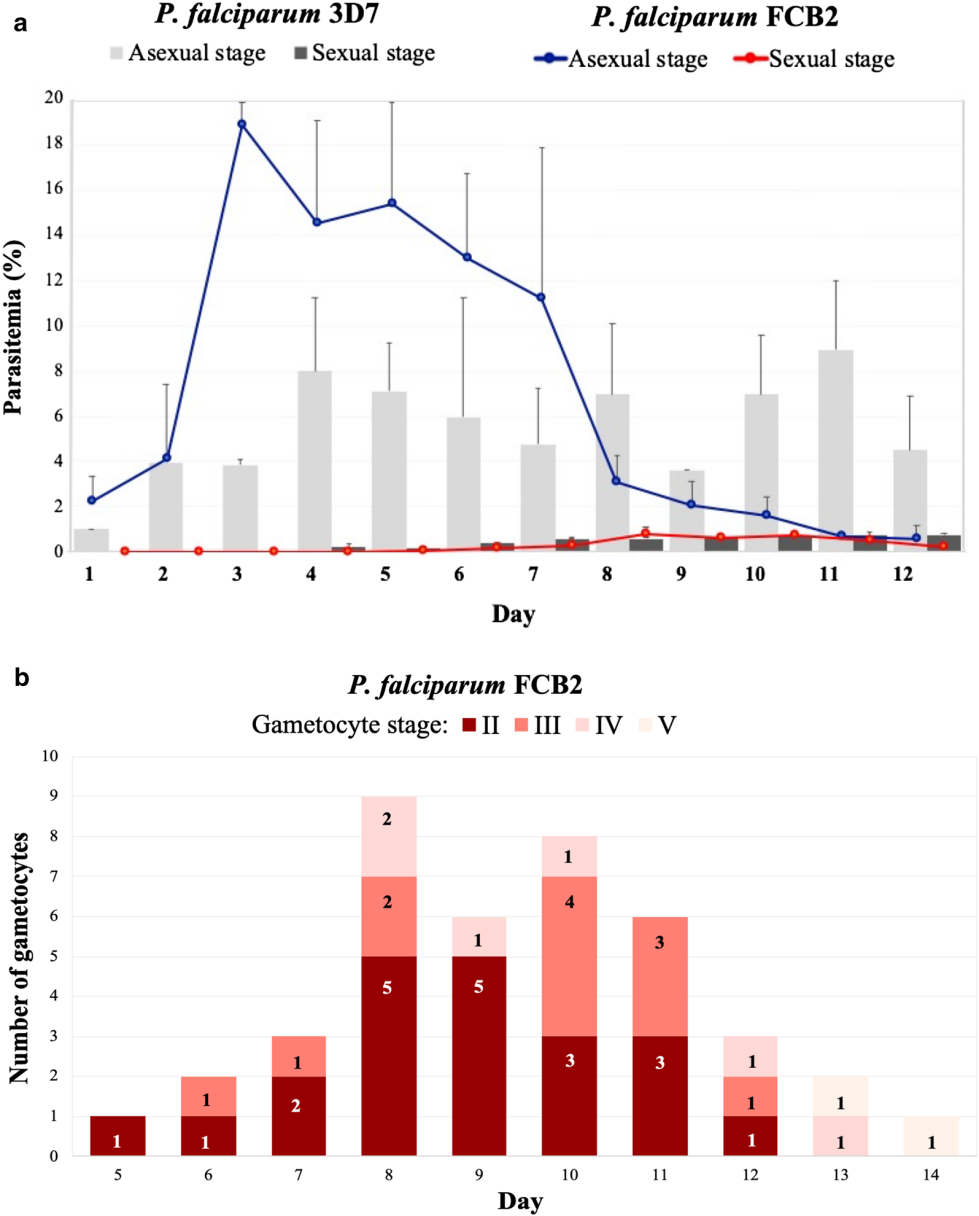
*Plasmodium falciparum* FCB2 and 3D7 gametocyte production. FCB2 and 3D7 strains’ gametocytes were obtained from parasite culture at 0.3% parasitaemia, analysed by Giemsa staining and parasitaemia and gametocyte stages were estimated by counting at 40X on a Primo Star Carl Zeiss microscope. 3 experimental replicates were made. **a** Relationship between FCB2 strain days’ culture and parasitaemia (Lines) and that for 3D7 strain (grey bars); **b** Number of FCB2 strain gametocytes at different stages according to the days in culture

*Pfap2g*, *Pfs16*, *Pfg27/25* and *Pfs25* gene transcription suggested the FCB2 strain’s sexual differentiation capability but did not prove gametocyte development. Morphological analysis of the forms produced by this parasite followed gametocyte production induction; such changes were evaluated for 12 to 15 days until the gametocytes reached their full mature morphology [22]. The parasites started to have clear morphological differentiation by day 4, beginning with a larger round shape which started to differentiate from the trophozoite forms. They then progressed to a D form before progressing to full mature gametocyte morphology from day 13 onwards (Figs. 2 and 3), as expected for gametocytogenesis [43]. FCB2 strain gametocytes conserved most of the expected features, having smooth and normal-sized parasite forms during each stage [44, 45] (Fig. 2).

FCB2 gametocyte production was analysed using the *P. falciparum* 3D7 strain as control. Sexual differentiation was induced in both in vitro cultured strains; the sexual forms obtained in each strain were counted, finding similar FCB2 strain gametocyte production (0.2% gametocytaemia) compared to that for the 3D7 strain (0.8% gametocytaemia) (Fig. 3a). A previous study reported maximum 0.8% 3D7 gametocytaemia, thereby supporting our findings [46].

**Fig. 4:**
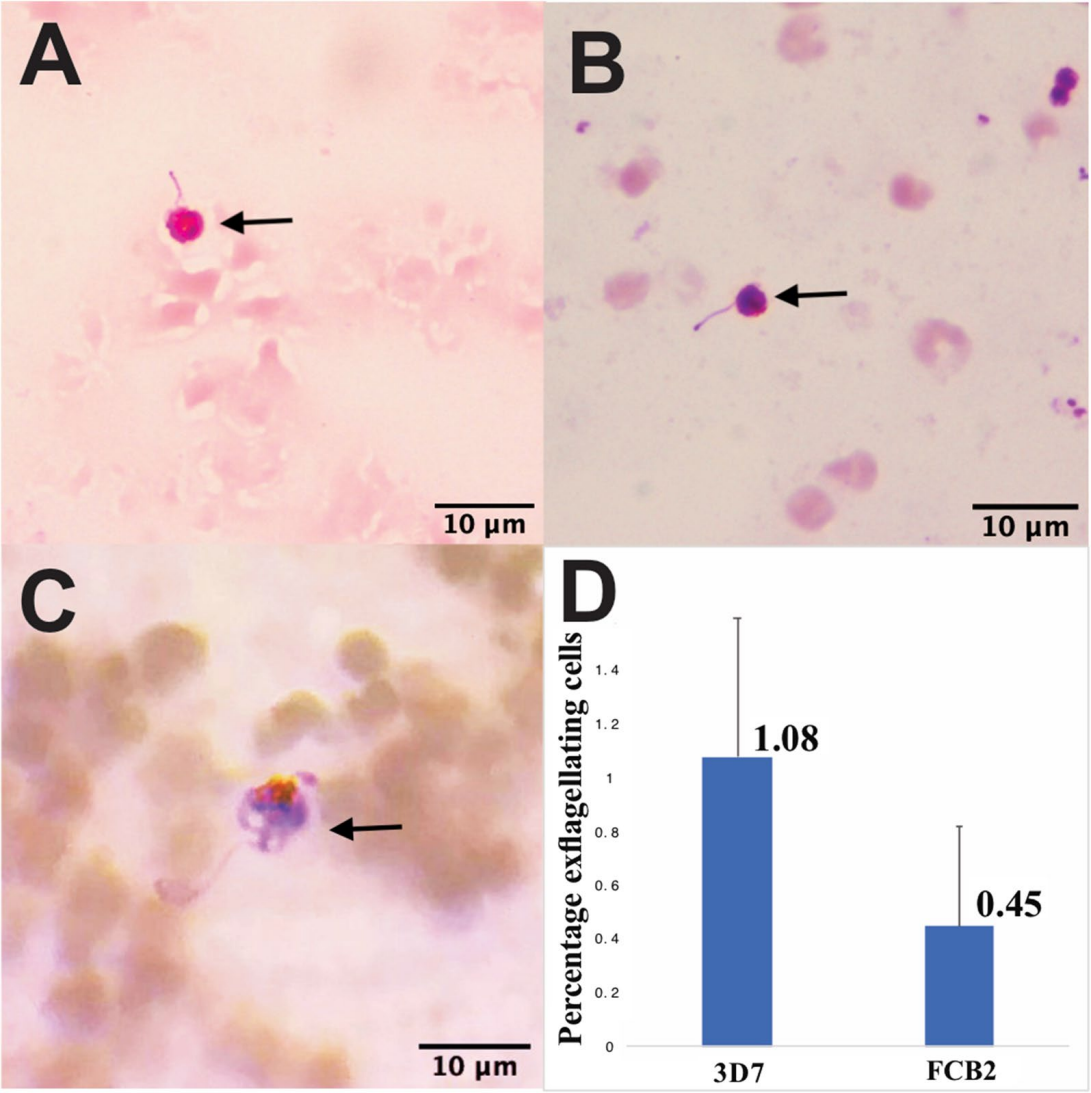
*Plasmodium falciparum* FCB2 exflagellation. FCB2 and 3D7 gametocyte culture exflagellation was induced by putting 50μl gametocyte culture sample at room temperature for 10 min. **A**–**C** FCB2 exflagellated microgametes; **D** exflagellation percentages (EP) were calculated for each strain (3D7 and FCB2); 3 experimental replicates were made. Exflagellated forms, analysed by Giemsa staining, were visualized at 100X on a Primo Star Carl Zeiss microscope

An exflagellation test was made after inducing sexual differentiation, finding 0.45% exflagellated cells in in vitro gametocyte culture, compared to 1.08% in 3D7 gametocyte culture (Fig. 4).

### *Plasmodium falciparum* FCB2 strain gametocyte infectivity

In vitro zygote formation was evaluated after placing FCB2 strain gametocyte culture samples in low temperature conditions (23 °C) to assess whether FCB2 strain gametocytes could infect mosquitoes. Round-shaped parasite forms (i.e. zygote forms) were observed, almost the same size as an erythrocyte (Fig. 2) [47]. Gametocyte infectivity was then analysed in vivo determining the amount of oocysts and their phenotypic characteristics in the mosquitoes’ midgut.

**Fig. 5:**
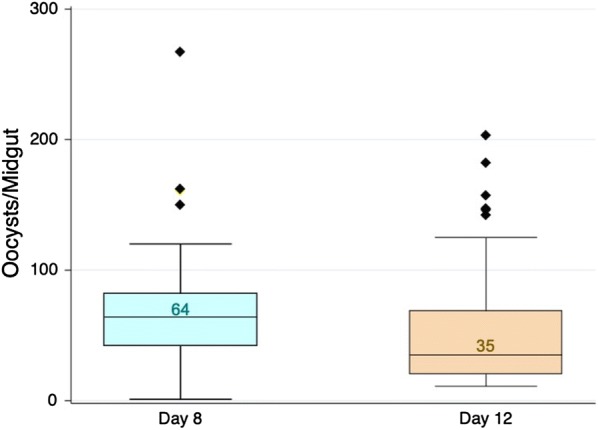
*Plasmodium falciparum* FCB2 oocyst production. FCB2 oocyst median production on day 9 and 12 post-infection in female mosquitoes infected with FCB2 gametocytes obtained from parasite culture at 0.3% parasitaemia were analysed by Giemsa staining

**Fig. 6:**
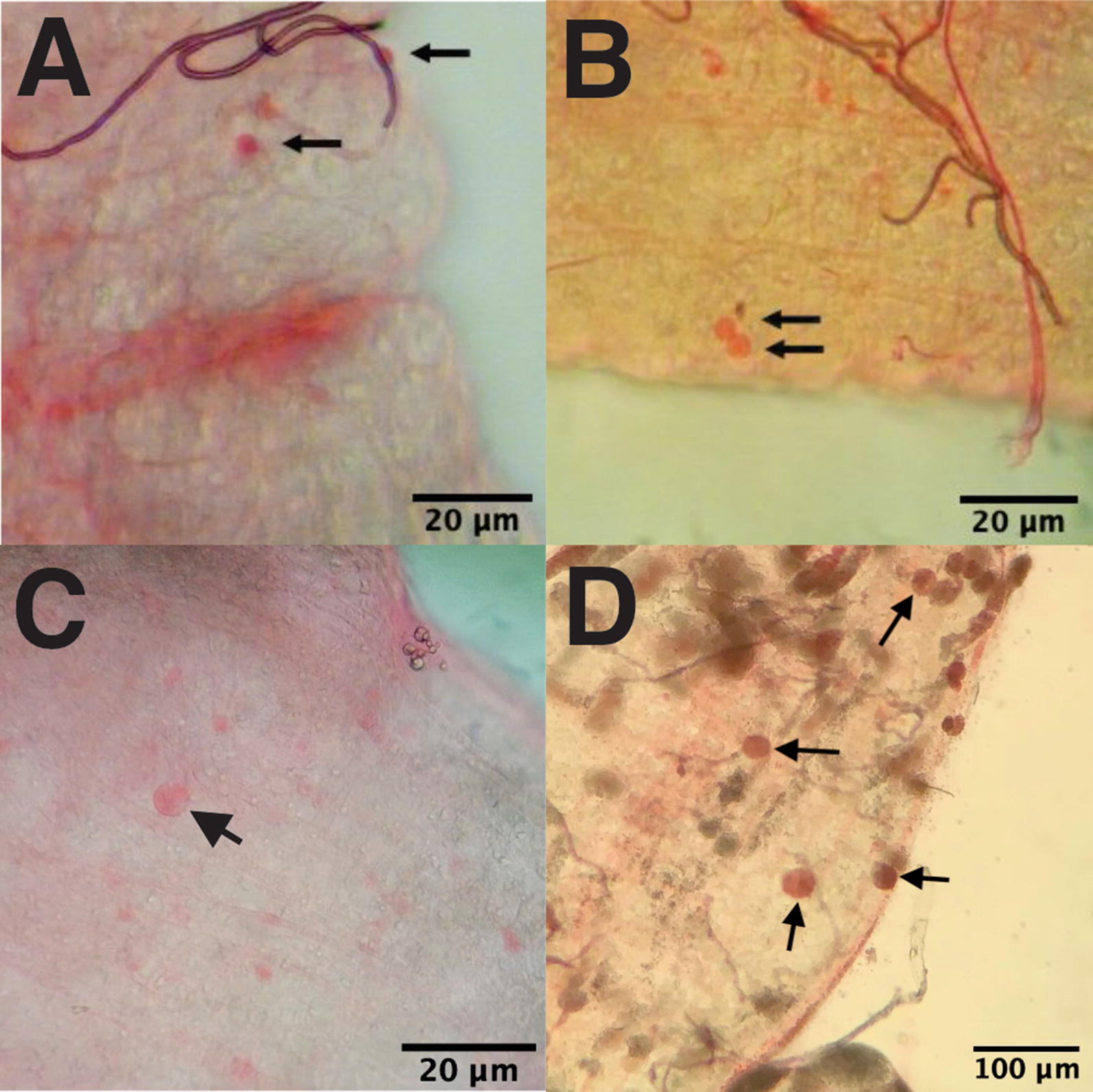
*Plasmodium falciparum* FCB2 oocyst morphological characteristics. Female mosquito midguts infected with FCB2 gametocytes obtained from parasite culture at 0.3% parasitaemia were analysed by 0.5% mercurochrome staining. **A** FCB2 oocysts day 8 post-infection; **B** FCB2 oocysts day 9 post-infection; **C** FCB2 oocysts day 12 post-infection; **D** FCB2 oocyst day 15 post-infection. All oocysts are indicated by black arrows, visualized at 10X and 40X on a Primo Star Carl Zeiss microscope

**Fig. 7:**
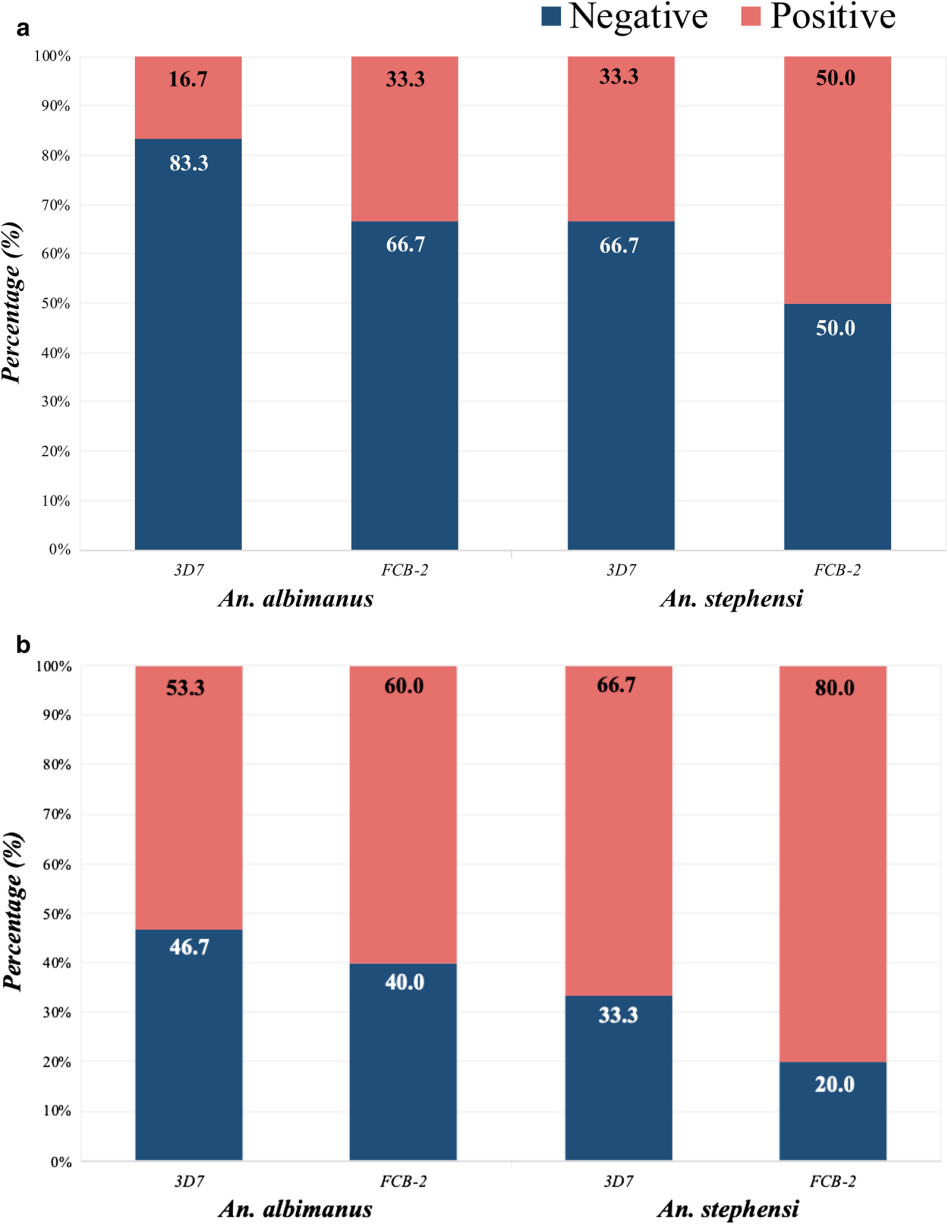
*Plasmodium falciparum* 3D7 and FCB2 strain oocyst production regarding mosquito species (*Anopheles albimanus* and *Anopheles stephensi*). Female mosquito midguts, infected with 3D7 and FCB2 strain gametocytes obtained from parasite culture at 0.3% parasitaemia were analysed by 0.5% mercurochrome staining. **a** 3D7 and FCB2 oocyst production on day 8 post-infection; **b** 3D7 and FCB2 oocyst production on day 12 post-infection

**Fig. 8:**
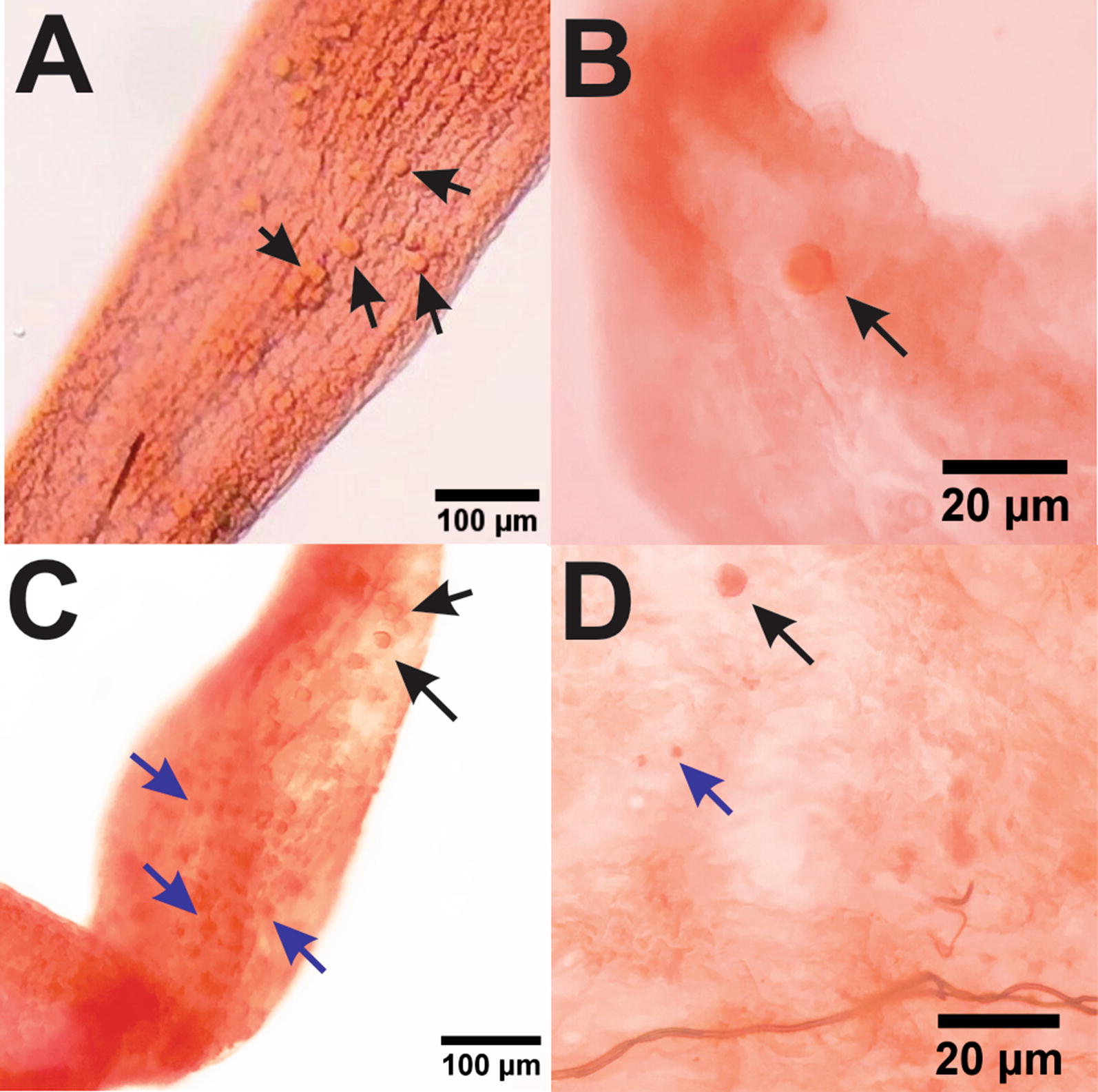
Comparing *Plasmodium falciparum* 3D7 and FCB2 strains’ oocyst morphological characteristics. Female mosquito midguts infected with FCB2 gametocytes obtained from parasite culture at 0.3% parasitaemia were analysed by 0.5% mercurochrome staining. **A**, **B** 3D7 oocysts on day 12 post-infection; **C**, **D** FCB2 oocysts on day 12 post-infection. Black arrows indicate viable oocysts; blue arrows show atrophic oocysts. All oocysts visualized at 40X on a Primo Star Carl Zeiss microscope

Female *An. albimanus* Buenaventura strain were fed with FCB2 gametocyte culture at 40% haematocrit, with human O+ erythrocytes. An initial FCB2 oocyst quantification in mosquito midgut until 10 to 12 days post-feeding show that the median value was higher on day 8 (median 64, interquartile range (IQR) 40) compared to day 12 (median 35, IQR 48); median distribution regarding the days evaluated here was statistically significant (*p *= 0.0147, *U* test) (Fig. 5). Oocyst development were followed by days 8, 9, 12 and also 15 post-feeding (Fig. 6). This was followed by oocyst detection in the mosquito midgut until 10 to 12 days post-feeding; the lowest amount of oocysts in *An. albimanus* midgut was observed on day 8 for 3D7 (16.7%) and FCB2 (33.3%), having no statistically significant difference between both strains (*p *= 0.833), compared to day 12 (53.3% for 3D7 and 60% for FCB2, *p *= 0.378) (Fig. 7). A similar pattern was seen for *An. stephensi* infection for 3D7 (33.3%) and FCB2 (50%) on day 8 (*p *= 0.800) and 3D7 (66.7%) and FCB2 (80%) on day 12 (*p *= 0.264) (Fig. 7). 3D7 and FCB2 strain oocysts’ morphological characteristics were compared. It was found that most FCB2 oocysts were similar to those from the 3D7 strain regarding their roundness and size, although many oocysts’ growth became stalled, indicating their atrophy (Fig. 8).

Even when the percentage of infected mosquito midguts was high on the 1st days studied, only 1.75% of mosquito females had normal oocyst development by day 12 (Figs. 6 and 8). Many factors affect parasite development inside a mosquito midgut, such as the mosquito’s immune response against the parasite (limiting *Plasmodium* growth and survival in mosquito midgut and haemolymph) [2, 48, 49]) and parasite mechanisms for evading mosquito immune systems (Pfs47-mediated [50, 51] and Pfs47 haplotype variation associated with its geographical origin [11]). Moreover, long-time FCB2 strain in vitro culture could contribute to such low oocyst maturation; it was thus hypothesized that a controlled in vitro environment may induce genomic and/or transcriptomic changes affecting gametocyte production and oocyst maturation. In-depth studies about genomic changes and transcriptional variation in oocyst growth-related genes must be made regarding the parasite’s sexual development in in vitro strains.

## Discussion

Malaria still remains a critical infectious disease because of the stalemate in controlling its progress since 2015 [1]. This problem has mostly been associated with the appearance of parasite resistance to anti-malarial treatment and the mosquito’s resistance to currently available insecticides [52, 53]. Asymptomatic patients (associated with silent host-vector transmission) are related to the disease’s epidemiological persistence thereby highlighting an increasing need for tools enabling the study of parasite transmissible forms [54–57].

As the parasite’s sexual forms are directly related to host-vector transmission, the in vitro study of antigens blocking this parasite stage is important for attacking this infection. Most studies usually involve using *P. falciparum* strains which have an already described differentiation capability [43, 58, 59]. Many studies use the NF54 parasite strain because of the ease of gametocyte production; some approaches in Latin-America have used the 7G8 strain [18, 19, 43]. Increasing the amount of characterized *P. falciparum* strains from other geographical regions having sexual differentiation ability might upgrade variability analysis and provide a better response to the need for anti-malarial drugs and vaccines.

The *P. falciparum* FCB2 strain was adapted from a severe malaria patient’s isolate and has been kept in in vitro culture for more than 30 years [24]. Most in vitro cultured parasite strains lose their sexual differentiation ability because of mutations in genes associated with the proteins needed for it, such as *Pfap2*-*g* [37]. This study has analysed the *P. falciparum* Colombian FCB2 strain’s sexual differentiation capability by initially verifying *Pfap2g*, *Pfs16*, *Pfg27/25* and *Pfs25* gene expression used for detecting infected patients who could have gametocytes and female parasite sexual forms (Fig. 1) [39–42, 60]. It is worth noting that the FCB2 strain has preserved its gametocyte production (although to a low degree: 0.2% gametocytes after 12 days culture) after more than 30 years of in vitro culture; it has conserved its characteristic phenotype during each gametocyte stage as seen in other sexually differentiated parasite strains (Fig. 2). These gametocytes were able to form zygotes and ookinetes and exflagellate after in vitro culture with low-temperature stimuli (Figs. 2, 3, 4).

The results highlighted the FCB2 strain’s differentiation ability and indicated its ability to infect mosquitoes. FCB2 strain gametocytes infected Colombian *An. albimanus* and *An. stephensi* using a controlled artificial mosquito feeding system; they differentiated into oocyst forms inside mosquito midgut, thereby confirming the conservation of the mosquitoes’ infection ability (Figs. 6 and 8). It is known that culture conditions influence gametocyte formation, i.e. erythrocyte percentage, hypoxanthine and glucose concentration [22, 61, 62]. Serum also influences gametocyte production; gametocyte production and their infectivity become decreased when using serum replacement substances [63]. Probably, such conserved FCB2 gametocyte production could have been associated with culture maintenance conditions, mostly related to parasite culture media always being supplemented with human plasma and this might have helped conserve this feature. Considering the implications of in vitro culture conditions regarding gametocytogenesis, it could be supposed that such conditions may also affect oocyst growth-associated genes, thereby causing impaired development of most of FCB2 oocysts. Genetic comparative analysis comparing high (e.g. NF54) and low (e.g. FCB2) oocyst-producing strains may help to resolve this question and also support the study of possible targets for anti-malarial drugs and vaccine development.

The FCB2 strain’s host geographical origin could also have influenced the amount of infected *An. albimanus* females and could have been related to the high percentage of oocysts recorded in this study. Mosquito infection potential studies regarding some parasite strains from different regions worldwide have shown that malarial transmission success directly depends on the geographical origin of mosquitoes and parasites [64–66]. However, further studies are needed (like the standard membrane-feeding assay using different strains) to confirm this hypothesis and confirming the compatibility between this strain and the geographic origin of *An. albimanus*.

A mosquito’s parasite infectivity is related to the parasite’s genetic factors enabling mosquitoes to avoid host innate immune response resulting from coevolution of both organisms [15]. Nevertheless, variation in mosquito infectivity has been reported when using *P. falciparum* isolates from the same geographical area, e.g. African strain NF54 infected 90% of *Anopheles gambiae*, Kenyan strain K39 infected 86% of *An. gambiae*, whilst M24 infected only 6% of the same mosquito species [15, 66]. Such huge difference regarding mosquito infection might be explained by variations in parasite strain infection susceptibility associated with mosquito immune response; specifically, increased *An. gambiae* thioester-containing protein 1 (TEP1) has been shown to be involved in oocyst killing, whilst parasite polymorphism in the *Pfs47* gene has enabled evading mosquito immune response [67].

Reports have shown that female *An. albimanus* infection with the 7G8 Brazilian strain was 68% and average oocyst production was 2 oocysts; such production was small compared to the FCB2 strain studied here (56 oocysts by day 12) and highlighted differences regarding compatibility between South American parasite strains and mosquitoes from the same region [11, 68]. Mosquito innate immune defence mechanisms may influence midgut epithelial ookinete invasion [69, 70]; mosquito immune responses could thus be related to the aforementioned study’s findings. Gametocytaemia, mosquito midgut xanthurenic acid concentration, haemozoin concentration, temperature and other intrinsic mosquito characteristics also influenced FCB2 sporogony formation inside *An. albimanus* midgut [36, 71]; this might explain the large amount of these forms found on initial days post-feeding compared to the small amount of such forms which finally developed. Moreover, the *Anopheline* late immune response against oocysts has been described in other mosquito species; haemocytes have been responsible for reduced parasite survival, using unknown mechanisms [72]. Studying haemocyte cell defence response in *An. albimanus* could be interesting for recognizing its cellular immunity.

## Conclusions

This study has thus proposed that the *P. falciparum* FCB2 strain could be a useful tool for gametocyte production and mosquito infection studies, thereby enabling antigen analysis and comparing anti-malarial drug and vaccine effectiveness. *Plasmodium falciparum* sexual differentiation and mosquito infection studies can facilitate the identification of parasite survival mechanisms inside *Anopheles*, parasite strain infection ability and further New World *Anopheline* malaria-transmission characterization studies.

## Supplementary information

**Additional file 1.** *Pfrh1* and *Pfmsp2* multiple alignment of FCB2 sequence to database reported sequences.

## Data Availability

The datasets used and/or analysed during the current study are available from the corresponding author on reasonable request.

## Abbreviations

WHO: World Health Organization
*Ap2*-*g*: Apetala 2-gametes
FCB2: *falciparum* Colombia Bogotá 2

## References

[1] WHO. World malaria report 2018. Geneva: World Health Organization; 2018. http://apps.who.int/iris/bitstream/handle/10665/275867/9789241565653-eng.pdf?ua=1. Accessed 25 Nov 2019.

[2] Crompton PD, Moebius J, Portugal S, Waisberg M, Hart G, Garver LS (2014). Malaria immunity in man and mosquito: insights into unsolved mysteries of a deadly infectious disease. Annu Rev Immunol.

[3] Cowman AF, Healer J, Marapana D, Marsh K (2016). Malaria: biology and disease. Cell.

[4] Molina-Cruz A, DeJong RJ, Ortega C, Haile A, Abban E, Rodrigues J (2012). Some strains of *Plasmodium falciparum*, a human malaria parasite, evade the complement-like system of *Anopheles gambiae* mosquitoes. Proc Natl Acad Sci USA.

[5] Baia-da-Silva DC, Alvarez LCS, Lizcano OV, Costa FTM, Lopes SCP, Orfanó AS (2018). The role of the peritrophic matrix and red blood cell concentration in *Plasmodium vivax* infection of *Anopheles aquasalis*. Parasit Vectors..

[6] Sauerwein RW, Bousema T (2015). Transmission blocking malaria vaccines: assays and candidates in clinical development. Vaccine..

[7] The malERA Consultative Group on Vaccines (2011). A research agenda for malaria eradication: vaccines. PLoS Med..

[8] Amoah LE, Acquah FK, Ayanful-Torgby R, Oppong A, Abankwa J, Obboh EK (2018). Dynamics of anti-MSP3 and Pfs230 antibody responses and multiplicity of infection in asymptomatic children from southern Ghana. Parasit Vectors..

[9] Blandin S, Shiao S-H, Moita LF, Janse CJ, Waters AP, Kafatos FC (2004). Complement-like protein TEP1 is a determinant of vectorial capacity in the malaria vector *Anopheles gambiae*. Cell.

[10] Bousema T, Roeffen W, Meijerink H, Mwerinde H, Mwakalinga S, van Gemert G-J (2010). The Dynamics of naturally acquired immune responses to *Plasmodium falciparum* sexual stage antigens Pfs230 & Pfs48/45 in a low endemic area in Tanzania. PLoS ONE.

[11] Molina-Cruz A, Canepa GE, Kamath N, Pavlovic NV, Mu J, Ramphul UN (2015). *Plasmodium* evasion of mosquito immunity and global malaria transmission: the lock-and-key theory. Proc Natl Acad Sci USA.

[12] Wu Y, Sinden RE, Churcher TS, Tsuboi T, Yusibov V (2015). Development of malaria transmission-blocking vaccines: from concept to product. Adv Parasitol.

[13] Miura K, Takashima E, Deng B, Tullo G, Diouf A, Moretz SE (2013). Functional comparison of *Plasmodium falciparum* transmission-blocking vaccine candidates by the standard membrane-feeding assay. Infect Immun.

[14] Smith RC, Barillas-Mury C, Jacobs-Lorena M (2015). Hemocyte differentiation mediates the mosquito late-phase immune response against *Plasmodium* in *Anopheles gambiae*. Proc Natl Acad Sci USA.

[15] Molina-Cruz A, Canepa GE, Barillas-Mury C (2017). Plasmodium P47: a key gene for malaria transmission by mosquito vectors. Curr Opin Microbiol.

[16] Garver LS, Bahia AC, Das S, Souza-Neto JA, Shiao J, Dong Y (2012). Anopheles Imd pathway factors and effectors in infection intensity-dependent anti-Plasmodium action. PLoS Pathog.

[17] Canepa GE, Molina-Cruz A, Barillas-Mury C (2016). Molecular analysis of Pfs47-mediated *Plasmodium* evasion of mosquito immunity. PLoS ONE.

[18] Pimenta PF, Orfano AS, Bahia AC, Duarte AP, Ríos-Velásquez CM, Melo FF (2015). An overview of malaria transmission from the perspective of Amazon *Anopheles* vectors. Mem Inst Oswaldo Cruz.

[19] Zapata JC, Perlaza BL, Hurtado S, Quintero GE, Jurado D, González I (2002). Reproducible infection of intact *Aotus lemurinus griseimembra* monkeys by *Plasmodium falciparum* sporozoite inoculation. J Parasitol.

[20] Olano VA, Carrillo MP, de la Vega P, Espinal CA (1985). Vector competence of Cartagena strain of *Anopheles albimanus* for *Plasmodium falciparum* and *P. vivax*. Trans R Soc Trop Med Hyg..

[21] Hurtado S, Salas ML, Romero JF, Zapata JC, Ortiz H, Arevalo-Herrera M (1997). Regular production of infective sporozoites of *Plasmodium falciparum* and *P. vivax* in laboratory-bred *Anopheles albimanus*. Ann Trop Med Parasitol..

[22] Delves MJ, Straschil U, Ruecker A, Miguel-Blanco C, Marques S, Dufour AC (2016). Routine in vitro culture of *P. falciparum* gametocytes to evaluate novel transmission-blocking interventions. Nat Protoc..

[23] Claessens A, Affara M, Assefa SA, Kwiatkowski DP, Conway DJ (2017). Culture adaptation of malaria parasites selects for convergent loss-of-function mutants. Sci Rep..

[24] Espinal TC, Moreno E, Guerra P, De La Vega P (1982). Aislamiento y caracterización de cepas colombiana de *Plasmodium falciparum*. Biomédica..

[25] Curtidor H, Vanegas M, Alba MP, Patarroyo ME (2011). Functional, immunological and three-dimensional analysis of chemically synthesised sporozoite peptides as components of a fully-effective antimalarial vaccine. Curr Med Chem.

[26] Rodriguez LE, Curtidor H, Urquiza M, Cifuentes G, Reyes C, Patarroyo ME (2008). Intimate molecular interactions of *P. falciparum* merozoite proteins involved in invasion of red blood cells and their implications for vaccine design. Chem Rev..

[27] Vásquez A, Segura C, Blair S (2013). Induction of pro-inflammatory response of the placental trophoblast by *Plasmodium falciparum* infected erythrocytes and TNF. Malar J..

[28] Malkin EM, Durbin AP, Diemert DJ, Sattabongkot J, Wu Y, Miura K (2005). Phase 1 vaccine trial of Pvs25H: a transmission blocking vaccine for *Plasmodium vivax* malaria. Vaccine..

[29] Wu Y, Ellis RD, Shaffer D, Fontes E, Malkin EM, Mahanty S (2008). Phase 1 Trial of malaria transmission blocking vaccine candidates Pfs25 and Pvs25 formulated with Montanide ISA 51. PLoS ONE.

[30] Trager W, Jensen JB (1976). Human malaria parasites in continuous culture. Science.

[31] Suaréz-Cortés P, Silvestrini F, Alano P (2014). A fast, non-invasive, quantitative staining protocol provides insights in *Plasmodium falciparum* gamete egress and in the role of osmiophilic bodies. Malar J..

[32] Osoga J, Waitumbi J, Guyah B, Sande J, Arima C, Ayaya M (2017). Comparative evaluation of fluorescent in situ hybridization and Giemsa microscopy with quantitative real-time PCR technique in detecting malaria parasites in a holoendemic region of Kenya. Malar J..

[33] Arévalo-Pinzón G, Curtidor H, Muñoz M, Suarez D, Patarroyo MA, Patarroyo ME (2013). Rh1 high activity binding peptides inhibit high percentages of *Plasmodium falciparum* FVO strain invasion. Vaccine..

[34] Larkin MA, Blackshields G, Brown NP, Chenna R, McGettigan PA, McWilliam H (2007). Clustal W and Clustal X version 2.0. Bioinformatics..

[35] Kennedy M, Fishbaugher ME, Vaughan AM, Patrapuvich R, Boonhok R, Yimamnuaychok N (2012). A rapid and scalable density gradient purification method for *Plasmodium* sporozoites. Malar J..

[36] Li T, Eappen AG, Richman AM, Billingsley PF, Abebe Y, Li M (2015). Robust, reproducible, industrialized, standard membrane feeding assay for assessing the transmission blocking activity of vaccines and drugs against *Plasmodium falciparum*. Malar J..

[37] Kafsack BFC, Rovira-Graells N, Clark TG, Bancells C, Crowley VM, Campino SG (2014). A transcriptional switch underlies commitment to sexual development in malaria parasites. Nature.

[38] Modrzynska K, Pfander C, Chappell L, Yu L, Suarez C, Dundas K (2017). A Knockout screen of ApiAP2 genes reveals networks of interacting transcriptional regulators controlling the *Plasmodium* life cycle. Cell Host Microbe.

[39] Alano P, Premawansa S, Bruce MC, Carter R (1991). A stage specific gene expressed at the onset of gametocytogenesis in *Plasmodium falciparum*. Mol Biochem Parasitol.

[40] Bousema T, Okell L, Felger I, Drakeley C (2014). Asymptomatic malaria infections: detectability, transmissibility and public health relevance. Nat Rev Microbiol.

[41] Kongkasuriyachai D, Fujioka H, Kumar N (2004). Functional analysis of *Plasmodium falciparum* parasitophorous vacuole membrane protein (Pfs16) during gametocytogenesis and gametogenesis by targeted gene disruption. Mol Biochem Parasitol.

[42] Wampfler R, Mwingira F, Javati S, Robinson L, Betuela I, Siba P (2013). Strategies for detection of *Plasmodium* species gametocytes. PLoS ONE.

[43] Talman AM, Domarle O, McKenzie FE, Ariey F, Robert V (2004). Gametocytogenesis: the puberty of *Plasmodium falciparum*. Malar J..

[44] Baker DA (2010). Malaria gametocytogenesis. Mol Biochem Parasitol.

[45] Bousema T, Drakeley C (2011). Epidemiology and infectivity of *Plasmodium falciparum* and *Plasmodium vivax* gametocytes in relation to malaria control and elimination. Clin Microbiol Rev.

[46] Gebru T, Lalremruata A, Kremsner PG, Mordmüller B, Held J (2017). Life-span of in vitro differentiated *Plasmodium falciparum* gametocytes. Malar J..

[47] Ghosh AK, Dinglasan RR, Ikadai H, Jacobs-Lorena M (2010). An improved method for the in vitro differentiation of *Plasmodium falciparum* gametocytes into ookinetes. Malar J..

[48] Bartholomay LC, Michel K (2018). Mosquito immunobiology: the intersection of vector health and vector competence. Annu Rev Entomol.

[49] Smith RC, Vega-Rodríguez J, Jacobs-Lorena M (2014). The *Plasmodium* bottleneck: malaria parasite losses in the mosquito vector. Mem Inst Oswaldo Cruz.

[50] Molina-Cruz A, Garver LS, Alabaster A, Bangiolo L, Haile A, Winikor J (2013). The human malaria parasite Pfs47 gene mediates evasion of the mosquito immune system. Science.

[51] Ramphul UN, Garver LS, Molina-Cruz A, Canepa GE, Barillas-Mury C (2015). *Plasmodium falciparum* evades mosquito immunity by disrupting JNK-mediated apoptosis of invaded midgut cells. Proc Natl Acad Sci USA.

[52] Amato R, Pearson RD, Almagro-Garcia J, Amaratunga C, Lim P, Suon S (2018). Origins of the current outbreak of multidrug-resistant malaria in southeast Asia: a retrospective genetic study. Lancet Infect Dis..

[53] Ranson H, Lissenden N (2016). Insecticide resistance in African Anopheles mosquitoes: a worsening situation that needs urgent action to maintain malaria control. Trends Parasitol..

[54] Hassanpour G, Mohebali M, Zeraati H, Raeisi A, Keshavarz H (2017). Asymptomatic malaria and its challenges in the malaria elimination program in Iran: a systematic review. J Arthropod-Borne Dis..

[55] Kapesa A, Kweka EJ, Atieli H, Afrane YA, Kamugisha E, Lee M-C (2018). The current malaria morbidity and mortality in different transmission settings in Western Kenya. PLoS ONE.

[56] Lin JT, Saunders DL, Meshnick SR (2014). The role of submicroscopic parasitemia in malaria transmission: what is the evidence?. Trends Parasitol..

[57] Vásquez-Jiménez JM, Arévalo-Herrera M, Henao-Giraldo J, Molina-Gómez K, Arce-Plata M, Vallejo AF (2016). Consistent prevalence of asymptomatic infections in malaria endemic populations in Colombia over time. Malar J..

[58] Ponnudurai T, Lensen AHW, Van Gemert GJA, Bensink MPE, Bolmer M, Meuwissen JHETh (1989). Infectivity of cultured *Plasmodium falciparum* gametocytes to mosquitoes. Parasitology.

[59] Itsara LS, Zhou Y, Do J, Dungel S, Fishbaugher ME, Betz WW (2018). PfCap380 as a marker for *Plasmodium falciparum* oocyst development in vivo and in vitro. Malar J..

[60] Kast K, Berens-Riha N, Zeynudin A, Abduselam N, Eshetu T, Löscher T (2013). Evaluation of *Plasmodium falciparum* gametocyte detection in different patient material. Malar J..

[61] Ifediba T, Vanderberg JP (1981). Complete in vitro maturation of *Plasmodium falciparum* gametocytes. Nature.

[62] Schuster FL (2002). Cultivation of *Plasmodium* spp. Clin Microbiol Rev.

[63] Lingnau A, Margos G, Maier WA, Seitz HM (1993). Serum-free cultivation of *Plasmodium falciparum* gametocytes in vitro. Parasitol Res.

[64] Baton LA, Ranford-Cartwright LC (2012). Ookinete destruction within the mosquito midgut lumen explains *Anopheles albimanus* refractoriness to *Plasmodium falciparum* (3D7A) oocyst infection. Int J Parasitol.

[65] Grieco JP, Achee NL, Roberts DR, Andre RG (2005). Comparative susceptibility of three species of *Anopheles* from Belize, Central America, to *Plasmodium falciparum* (NF-54). J Am Mosq Control Assoc..

[66] Hume JC, Tunnicliff M, Ranford-Cartwright LC, Day KP (2007). Susceptibility of *Anopheles gambiae* and *Anopheles stephensi* to tropical isolates of *Plasmodium falciparum*. Malar J..

[67] Eldering M, Morlais I, van Gemert G-J, van de Vegte-Bolmer M, Graumans W, Siebelink-Stoter R (2016). Variation in susceptibility of African *Plasmodium falciparum* malaria parasites to TEP1 mediated killing in *Anopheles gambiae* mosquitoes. Sci Rep..

[68] Orfano AS, Duarte APM, Molina-Cruz A, Pimenta PF, Barillas-Mury C (2016). *Plasmodium yoelii nigeriensis* (N67) is a robust animal model to study malaria transmission by South American Anopheline mosquitoes. PLoS ONE.

[69] Jaramillo-Gutierrez G, Rodrigues J, Ndikuyeze G, Povelones M, Molina-Cruz A, Barillas-Mury C (2009). Mosquito immune responses and compatibility between *Plasmodium* parasites and anopheline mosquitoes. BMC Microbiol.

[70] Shaw WR, Catteruccia F (2019). Vector biology meets disease control: using basic research to fight vector-borne diseases. Nat Microbiol..

[71] Hauck ES, Antonova-Koch Y, Drexler A, Pietri J, Pakpour N, Liu D (2013). Overexpression of phosphatase and tensin homolog improves fitness and decreases *Plasmodium falciparum* development in *Anopheles stephensi*. Microbes Infect.

[72] Smith RC, Barillas-Mury C (2016). *Plasmodium* oocysts: overlooked targets of mosquito immunity. Trends Parasitol..

