# Supplementary material for: Sexual forms obtained in a continuous in vitro cultured Colombian strain of Plasmodium falciparum (FCB2)
Source: Malar J. 2020 Feb 3;19:57. doi: 10.1186/s12936-020-3142-y (PMC6998264; doi:10.1186/s12936-020-3142-y)
Supplement: Supplementary file 1 — Additional file 1. Pfrh1 and Pfmsp2 multiple alignment of FCB2 sequence to database reported sequences. [file 12936_2020_3142_MOESM1_ESM.docx]

**Additional file 1.** *Pfrh1* and *Pfmsp2* multiple alignment of FCB2 sequence to database reported sequences

*Pfrh1*

| Reference sequence (1): 3D7  Identities normalised by aligned length.  Colored by: identity |
| --- |
| cov pid  **1** **[ . . . . : . . .** **80**  1 3D7 100.0% 100.0% **ACAAATAAAATAAATACACATAATAAGAAGAACCAAGAAATGATGGAAGAATTCATATATGCATATAAAAGGTTAAAAAT**  2 NF54 100.0% 100.0% **ACAAATAAAATAAATACACATAATAAGAAGAACCAAGAAATGATGGAAGAATTCATATATGCATATAAAAGGTTAAAAAT**  3 HB3 100.0% 100.0% **ACAAATAAAATAAATACACATAATAAGAAGAACCAAGAAATGATGGAAGAATTCATATATGCATATAAAAGGTTAAAAAT**  4 FCB2 100.0% 99.8% **ACAAATAAAATAAATACACATAATAAGAAGAACCAAGAAATGATGGAAGAATTCATATATGTATATAAAAGGTTAAAAAT**  5 FVO 100.0% 99.8% **ACAAATAAAATAAATACACATAATAAGAAGAACCAAGAAATGATGGAAGAATTCATATATGTATATAAAAGGTTAAAAAT**  6 CAMP/Malaysia 100.0% 99.7% **ACAAATAAAATAAATACACATAATAAGAAGAACCAAGAAATGATGGAAGAATTCATATATGTATATAAAAGGTTAAAAAT**  7 Senegal_V34.04 100.0% 99.7% **ACAAATAAAATAAATACACATAATAAGAAGAACCAAGAAATGATGGAAGAATTCATATATGCATATAAAAGGTTAAAAAT**  8 D10 100.0% 99.5% **ACAAATAAAATAAATACACATAATAAGAAGAACCAAGAAATGATGGAAGAATTCATATATGTATATAAAAGGTTAAAAAT**  consensus/100% **ACAAATAAAATAAATACACATAATAAGAAGAACCAAGAAATGATGGAAGAATTCATATATGsATATAAAAGGTTAAAAAT**  consensus/90% **ACAAATAAAATAAATACACATAATAAGAAGAACCAAGAAATGATGGAAGAATTCATATATGsATATAAAAGGTTAAAAAT**  consensus/80% **ACAAATAAAATAAATACACATAATAAGAAGAACCAAGAAATGATGGAAGAATTCATATATGsATATAAAAGGTTAAAAAT**  consensus/70% **ACAAATAAAATAAATACACATAATAAGAAGAACCAAGAAATGATGGAAGAATTCATATATGsATATAAAAGGTTAAAAAT**  cov pid  **81**  **. 1 . . . . : .** **160**  1 3D7 100.0% 100.0% **TTTAAAAATATTAAATATATCCTTAAAAGCTTGTGAAAAAAATAATAAATCTATCAATACATTAAATGACAAAACACAAG**  2 NF54 100.0% 100.0% **TTTAAAAATATTAAATATATCCTTAAAAGCTTGTGAAAAAAATAATAAATCTATCAATACATTAAATGACAAAACACAAG**  3 HB3 100.0% 100.0% **TTTAAAAATATTAAATATATCCTTAAAAGCTTGTGAAAAAAATAATAAATCTATCAATACATTAAATGACAAAACACAAG**  4 FCB2 100.0% 99.8% **TTTAAAAATATTAAATATATCCTTAAAAGCTTGTGAAAAAAATAATAAATCTATCAATACATTAAATGACAAAACACAAG**  5 FVO 100.0% 99.8% **TTTAAAAATATTAAATATATCCTTAAAAGCTTGTGAAAAAAATAATAAATCTATCAATACATTAAATGACAAAACACAAG**  6 CAMP/Malaysia 100.0% 99.7% **TTTTAAAATATTAAATATATCCTTAAAAGCTTGTGAAAAAAATAATAAATCTATCAATACATTAAATGACAAAACACAAG**  7 Senegal_V34.04 100.0% 99.7% **TTTTAAAATATTAAATATATCCTTAAAAGCTTGTGAAAAAAATAATAAATCTATCAATACATTAAATGACAAAACACAAG**  8 D10 100.0% 99.5% **TTTTAAAATATTAAATATATCCTTAAAAGCTTGTGAAAAAAATAATAAATCTATCAATACATTAAATGACAAAACACAAG**  consensus/100% **TTTsAAAATATTAAATATATCCTTAAAAGCTTGTGAAAAAAATAATAAATCTATCAATACATTAAATGACAAAACACAAG**  consensus/90% **TTTsAAAATATTAAATATATCCTTAAAAGCTTGTGAAAAAAATAATAAATCTATCAATACATTAAATGACAAAACACAAG**  consensus/80% **TTTsAAAATATTAAATATATCCTTAAAAGCTTGTGAAAAAAATAATAAATCTATCAATACATTAAATGACAAAACACAAG**  consensus/70% **TTTsAAAATATTAAATATATCCTTAAAAGCTTGTGAAAAAAATAATAAATCTATCAATACATTAAATGACAAAACACAAG**  cov pid **161**  **. . . 2 . . . .** **240**  1 3D7 100.0% 100.0% **AATTAAAAAAAATTGTAACACACGAAATAGATCTTCTACAAAAAGATATTTTAACAAGTCAAATATCAAATAAAAATGTT**  2 NF54 100.0% 100.0% **AATTAAAAAAAATTGTAACACACGAAATAGATCTTCTACAAAAAGATATTTTAACAAGTCAAATATCAAATAAAAATGTT**  3 HB3 100.0% 100.0% **AATTAAAAAAAATTGTAACACACGAAATAGATCTTCTACAAAAAGATATTTTAACAAGTCAAATATCAAATAAAAATGTT**  4 FCB2 100.0% 99.8% **AATTAAAAAAAATTGTAACACACGAAATAGATCTTCTACAAAAAGATATTTTAACAAGTCAAATATCAAATAAAAATGTT**  5 FVO 100.0% 99.8% **AATTAAAAAAAATTGTAACACACGAAATAGATCTTCTACAAAAAGATATTTTAACAAGTCAAATATCAAATAAAAATGTT**  6 CAMP/Malaysia 100.0% 99.7% **AATTAAAAAAAATTGTAACACACGAAATAGATCTTCTACAAAAAGATATTTTAACAAGTCAAATATCAAATAAAAATGTT**  7 Senegal_V34.04 100.0% 99.7% **AATTAAAAAAAATTGTAACACACGAAATAGATCTTCTACAAAAAGATATTTTAACAAGTCAAATATCAAATAAAAATGTT**  8 D10 100.0% 99.5% **AATTAAAAAAAATTGTAACACACGAAATAGATCTTCTACAAAAAGATATTTTAACAAGTCAAATATCAAATAAAAATGTT**  consensus/100% **AATTAAAAAAAATTGTAACACACGAAATAGATCTTCTACAAAAAGATATTTTAACAAGTCAAATATCAAATAAAAATGTT**  consensus/90% **AATTAAAAAAAATTGTAACACACGAAATAGATCTTCTACAAAAAGATATTTTAACAAGTCAAATATCAAATAAAAATGTT**  consensus/80% **AATTAAAAAAAATTGTAACACACGAAATAGATCTTCTACAAAAAGATATTTTAACAAGTCAAATATCAAATAAAAATGTT**  consensus/70% **AATTAAAAAAAATTGTAACACACGAAATAGATCTTCTACAAAAAGATATTTTAACAAGTCAAATATCAAATAAAAATGTT**  cov pid **241**  **: . . . . 3 . .** **320**  1 3D7 100.0% 100.0% **TTATTATTAAACGATTTATTAAAAGAAATTGAACAATATATTATAGATGTACACAAATTAAAAAAAAAATCAAACGATCT**  2 NF54 100.0% 100.0% **TTATTATTAAACGATTTATTAAAAGAAATTGAACAATATATTATAGATGTACACAAATTAAAAAAAAAATCAAACGATCT**  3 HB3 100.0% 100.0% **TTATTATTAAACGATTTATTAAAAGAAATTGAACAATATATTATAGATGTACACAAATTAAAAAAAAAATCAAACGATCT**  4 FCB2 100.0% 99.8% **TTATTATTAAACGATTTATTAAAAGAAATTGAACAATATATTATAGATGTACACAAATTAAAAAAAAAATCAAACGATCT**  5 FVO 100.0% 99.8% **TTATTATTAAACGATTTATTAAAAGAAATTGAACAATATATTATAGATGTACACAAATTAAAAAAAAAATCAAACGATCT**  6 CAMP/Malaysia 100.0% 99.7% **TTATTATTAAACGATTTATTAAAAGAAATTGAACAATATATTATAGATGTACACAAATTAAAAAAAAAATCAAACGATCT**  7 Senegal_V34.04 100.0% 99.7% **TTATTATTAAACGATTTATTAAAAGAAATTGAACAATATATTATAGATATACACAAATTAAAAAAAAAATCAAACGATCT**  8 D10 100.0% 99.5% **TTATTATTAAACGATTTATTAAAAGAAATTGAACAATATATTATAGATATACACAAATTAAAAAAAAAATCAAACGATCT**  consensus/100% **TTATTATTAAACGATTTATTAAAAGAAATTGAACAATATATTATAGATuTACACAAATTAAAAAAAAAATCAAACGATCT**  consensus/90% **TTATTATTAAACGATTTATTAAAAGAAATTGAACAATATATTATAGATuTACACAAATTAAAAAAAAAATCAAACGATCT**  consensus/80% **TTATTATTAAACGATTTATTAAAAGAAATTGAACAATATATTATAGATuTACACAAATTAAAAAAAAAATCAAACGATCT**  consensus/70% **TTATTATTAAACGATTTATTAAAAGAAATTGAACAATATATTATAGATGTACACAAATTAAAAAAAAAATCAAACGATCT**  cov pid **321**  **. . : . . . . 4** **400**  1 3D7 100.0% 100.0% **ATTTACATATTATGAACAATCCAAAAATTATTTCTATTTTAAAAACAAAAAAGATAATTTTGATATACAAAAAACAATCA**  2 NF54 100.0% 100.0% **ATTTACATATTATGAACAATCCAAAAATTATTTCTATTTTAAAAACAAAAAAGATAATTTTGATATACAAAAAACAATCA**  3 HB3 100.0% 100.0% **ATTTACATATTATGAACAATCCAAAAATTATTTCTATTTTAAAAACAAAAAAGATAATTTTGATATACAAAAAACAATCA**  4 FCB2 100.0% 99.8% **ATTTACATATTATGAACAATCCAAAAATTATTTCTATTTTAAAAACAAAAAAGATAATTTTGATATACAAAAAACAATCA**  5 FVO 100.0% 99.8% **ATTTACATATTATGAACAATCCAAAAATTATTTCTATTTTAAAAACAAAAAAGATAATTTTGATATACAAAAAACAATCA**  6 CAMP/Malaysia 100.0% 99.7% **ATTTACATATTATGAACAATCCAAAAATTATTTCTATTTTAAAAACAAAAAAGATAATTTTGATATACAAAAAACAATCA**  7 Senegal_V34.04 100.0% 99.7% **ATTTACATATTATGAACAATCCAAAAATTATTTCTATTTTAAAAACAAAAAAGATAATTTTGATATACAAAAAACAATCA**  8 D10 100.0% 99.5% **ATTTACATATTATGAACAATCCAAAAATTATTTCTATTTTAAAAACAAAAAAGATAATTTTGATATACAAAAAACAATCA**  consensus/100% **ATTTACATATTATGAACAATCCAAAAATTATTTCTATTTTAAAAACAAAAAAGATAATTTTGATATACAAAAAACAATCA**  consensus/90% **ATTTACATATTATGAACAATCCAAAAATTATTTCTATTTTAAAAACAAAAAAGATAATTTTGATATACAAAAAACAATCA**  consensus/80% **ATTTACATATTATGAACAATCCAAAAATTATTTCTATTTTAAAAACAAAAAAGATAATTTTGATATACAAAAAACAATCA**  consensus/70% **ATTTACATATTATGAACAATCCAAAAATTATTTCTATTTTAAAAACAAAAAAGATAATTTTGATATACAAAAAACAATCA**  cov pid **401**  **. . . . : . . .** **480**  1 3D7 100.0% 100.0% **ATAAAATGAATGAATGGCTAGCTATCAAAAATTATATAAATGAAATTAATAAAAATTATCAAACATTATATGAAAAAAAA**  2 NF54 100.0% 100.0% **ATAAAATGAATGAATGGCTAGCTATCAAAAATTATATAAATGAAATTAATAAAAATTATCAAACATTATATGAAAAAAAA**  3 HB3 100.0% 100.0% **ATAAAATGAATGAATGGCTAGCTATCAAAAATTATATAAATGAAATTAATAAAAATTATCAAACATTATATGAAAAAAAA**  4 FCB2 100.0% 99.8% **ATAAAATGAATGAATGGCTAGCTATCAAAAATTATATAAATGAAATTAATAAAAATTATCAAACATTATATGAAAAAAAA**  5 FVO 100.0% 99.8% **ATAAAATGAATGAATGGCTAGCTATCAAAAATTATATAAATGAAATTAATAAAAATTATCAAACATTATATGAAAAAAAA**  6 CAMP/Malaysia 100.0% 99.7% **ATAAAATGAATGAATGGCTAGCTATCAAAAATTATATAAATGAAATTAATAAAAATTATCAAACATTATATGAAAAAAAA**  7 Senegal_V34.04 100.0% 99.7% **ATAAAATGAATGAATGGCTAGCTATCAAAAATTATATAAATGAAATTAATAAAAATTATCAAACATTATATGAAAAAAAA**  8 D10 100.0% 99.5% **ATAAAATGAATGAATGGCTAGCTATCAAAAATTATATAAATGAAATTAATAAAAATTATCAAACATTATATGAAAAAAAA**  consensus/100% **ATAAAATGAATGAATGGCTAGCTATCAAAAATTATATAAATGAAATTAATAAAAATTATCAAACATTATATGAAAAAAAA**  consensus/90% **ATAAAATGAATGAATGGCTAGCTATCAAAAATTATATAAATGAAATTAATAAAAATTATCAAACATTATATGAAAAAAAA**  consensus/80% **ATAAAATGAATGAATGGCTAGCTATCAAAAATTATATAAATGAAATTAATAAAAATTATCAAACATTATATGAAAAAAAA**  consensus/70% **ATAAAATGAATGAATGGCTAGCTATCAAAAATTATATAAATGAAATTAATAAAAATTATCAAACATTATATGAAAAAAAA**  cov pid **481**  **. 5 . . . . : .** **560**  1 3D7 100.0% 100.0% **ATAAATGTACTCCTACATAATTCAAAAAGTTATGTACAATACTTTTATGATCATATAATAAATCTAATTCTTCAAAAAAA**  2 NF54 100.0% 100.0% **ATAAATGTACTCCTACATAATTCAAAAAGTTATGTACAATACTTTTATGATCATATAATAAATCTAATTCTTCAAAAAAA**  3 HB3 100.0% 100.0% **ATAAATGTACTCCTACATAATTCAAAAAGTTATGTACAATACTTTTATGATCATATAATAAATCTAATTCTTCAAAAAAA**  4 FCB2 100.0% 99.8% **ATAAATGTACTCCTACATAATTCAAAAAGTTATGTACAATACTTTTATGATCATATAATAAATCTAATTCTTCAAAAAAA**  5 FVO 100.0% 99.8% **ATAAATGTACTCCTACATAATTCAAAAAGTTATGTACAATACTTTTATGATCATATAATAAATCTAATTCTTCAAAAAAA**  6 CAMP/Malaysia 100.0% 99.7% **ATAAATGTACTCCTACATAATTCAAAAAGTTATGTACAATACTTTTATGATCATATAATAAATCTAATTCTTCAAAAAAA**  7 Senegal_V34.04 100.0% 99.7% **ATAAATGTACTCCTACATAATTCAAAAAGTTATGTACAATACTTTTATGATCATATAATAAATCTAATTCTTCAAAAAAA**  8 D10 100.0% 99.5% **ATAAATGTACTCCTACATAATTCAAAAAGTTATGTACAATACTTTTATGATCATATAATAAATCTAATTCTTCAAAAAAA**  consensus/100% **ATAAATGTACTCCTACATAATTCAAAAAGTTATGTACAATACTTTTATGATCATATAATAAATCTAATTCTTCAAAAAAA**  consensus/90% **ATAAATGTACTCCTACATAATTCAAAAAGTTATGTACAATACTTTTATGATCATATAATAAATCTAATTCTTCAAAAAAA**  consensus/80% **ATAAATGTACTCCTACATAATTCAAAAAGTTATGTACAATACTTTTATGATCATATAATAAATCTAATTCTTCAAAAAAA**  consensus/70% **ATAAATGTACTCCTACATAATTCAAAAAGTTATGTACAATACTTTTATGATCATATAATAAATCTAATTCTTCAAAAAAA**  cov pid **561**  **. . . ]** **598**  1 3D7 100.0% 100.0% **AAATTATTTGGAAAATACTTTAAAGACAAAAATACAAG**  2 NF54 100.0% 100.0% **AAATTATTTGGAAAATACTTTAAAGACAAAAATACAAG**  3 HB3 100.0% 100.0% **AAATTATTTGGAAAATACTTTAAAGACAAAAATACAAG**  4 FCB2 100.0% 99.8% **AAATTATTTGGAAAATACTTTAAAGACAAAAATACAAG**  5 FVO 100.0% 99.8% **AAATTATTTGGAAAATACTTTAAAGACAAAAATACAAG**  6 CAMP/Malaysia 100.0% 99.7% **AAATTATTTGGAAAATACTTTAAAGACAAAAATACAAG**  7 Senegal_V34.04 100.0% 99.7% **AAATTATTTGGAAAATACTTTAAAGACAAAAATACAAG**  8 D10 100.0% 99.5% **AAATTATTTGGAAAATACTTTAAAGACAAAAATACAAG**  consensus/100% **AAATTATTTGGAAAATACTTTAAAGACAAAAATACAAG**  consensus/90% **AAATTATTTGGAAAATACTTTAAAGACAAAAATACAAG**  consensus/80% **AAATTATTTGGAAAATACTTTAAAGACAAAAATACAAG**  consensus/70% **AAATTATTTGGAAAATACTTTAAAGACAAAAATACAAG** |

*Pfmsp2*

| Reference sequence (1): FCB2_msp2  Identities normalised by aligned length.  Colored by: identity |
| --- |
| cov pid  **1** **[ . . . . : . . .** **80**  1 FCB2_msp2 100.0% 100.0% **-------------------------------------------------------------------GCAACACAGATTA**  2 3D7 63.6% 65.9% **AAAACATTGTCTATTATAAATTTCTTTATTTTTGTTACCTTTAATATTAAAAATGAAAGTAAATATAGCAACACA---TT**  3 7G8 48.5% 88.3% **-------------------------------------------------------------------GCAACACA---TT**  4 FVO 74.9% 33.7% **--------------------------------------------------------------------------------**  5 NF54 68.2% 40.1% **------------------------------------------------------------------ACCATCAC------**  6 CAMP/Malaysia 62.8% 38.2% **--------------------------------------------------------------------------------**  7 HB3 86.6% 36.4% **--------------------------------------------------------------------------------**  consensus/100% **................................................................................**  consensus/90% **................................................................................**  consensus/80% **................................................................................**  consensus/70% **................................................................................**  cov pid  **81**  **. 1 . . . . : .** **160**  1 FCB2_msp2 100.0% 100.0% **CATAAACAATGCTTATAATATGAGTATAAGGAGAAGTATGACAGA-------------AAAGTAATCCTCCTACTGGTGC**  2 3D7 63.6% 65.9% **CATAAACAATGCTTATAATATGAGTATAAGGAGAAGTATGGCAG--------------AAAGTAAGCCTTCTACTGGTGC**  3 7G8 48.5% 88.3% **CATAAACAATGCTTATAATATGAGTATAAGGAGAAGTATGGCAG--------------AAAGTAATCCTTCTACTGGTGC**  4 FVO 74.9% 33.7% **----------------------------------------ACCA--------------GCACTACCACCAGCACTACCAC**  5 NF54 68.2% 40.1% **CAGAACCAGCACTACCACCAGCACTACCACCAGCACTACCACCA--------------GCACTACCACCAGCACTACCAC**  6 CAMP/Malaysia 62.8% 38.2% **------CAGCATCTGCACCAGGATTAGCACCATTACCAGCACTA--------------GCACCAGCACCAGAACCAGCAC**  7 HB3 86.6% 36.4% **---------------TTATATGAATATGGCAAAAGATAAAACAAGTGTTGCTGAAATTAAAACAACAAATTTATTTATTG**  consensus/100% **........................................uCsu..............usAssAssssssssAsssssss**  consensus/90% **........................................uCsu..............usAssAssssssssAsssssss**  consensus/80% **...............ssssAssAsTAssussAssussAssuCsu..............usAssAsssCssssACTussuC**  consensus/70% **......CAususssssAssAsGAsTAssAssAusAsTAssACsu..............usAsTAussCssssACTuusuC**  cov pid **161**  **. . . 2 . . . .** **240**  1 FCB2_msp2 100.0% 100.0% **TAGTGGTAGTGCTGGTGGTAGTGCTGGTGGTAGTGCTGGTGGTAGTGCTGGTGGTAGTGCTGGTGGTAGTGCTGGTGGTA**  2 3D7 63.6% 65.9% **TGGTGGTAGTGCTGGTGGTAGTGCTGGTGGTAGTGCTGGTGGTAGTGCTGGTGGTAGTGCTGGTGGTAGTGCTGGT----**  3 7G8 48.5% 88.3% **TGGTGGTAGTGGTAGTGCTGGTGGTAGTGGTAGTGCTGGT----------------------------------------**  4 FVO 74.9% 33.7% **CAGCACTACCACTAGCACCAGTAGGAGGATTACTTTCTGTCATACTTCTCCTTATACTCATATTATAAGCATTGTTTA--**  5 NF54 68.2% 40.1% **CAGCACTACCACCAGCACCAGTAGAAGGCTTACTTTCTGCCATACTTCTCCTTATACTCATATTATAAGCATTGTTTA--**  6 CAMP/Malaysia 62.8% 38.2% **TACCACTACCACCAGTACCAGTAGGAGGCTTACTTTCTGCCATACTTCTCCTTATACTCATATTATAAGCATTGTTTA--**  7 HB3 86.6% 36.4% **AAGCAATATTACTAGAGTTATTTAAGAGGGATGTTGCTGCTCCACAGTTTTCTTTGTTACCATCGGTACATTCTTTTTGA**  consensus/100% **sussusTAssussuGsussusTsssuussssssTssssGs........................................**  consensus/90% **sussusTAssussuGsussusTsssuussssssTssssGs........................................**  consensus/80% **suGsusTAssuCsuGsussAGTussuGsssTAsTssssGssssAssssTsssssTusTsssussussAsssssssT....**  consensus/70% **sAGsusTAssuCTAGsussAGTuusuGsusTAsTssssGssuTAsTsCTssTsuTAsTssTusTussAGsusTGsTss..**  cov pid **241**  **: . . . . 3 . .** **320**  1 FCB2_msp2 100.0% 100.0% **GTGCTGGTGGTACTGCTGGTGGTACTGCTGGTGCTACCACTAGCACCA------------GTGCTGGAGGTATTACTTTC**  2 3D7 63.6% 65.9% **--------------------------------------------------------------------------------**  3 7G8 48.5% 88.3% **--------------------------------------------------------------------------------**  4 FVO 74.9% 33.7% **----TGAATGTGTTGCTATATTTACTTTCATTTTTAATATTAAAGGTAACAAAAATAAAGAAATTTATAATAGACAATGT**  5 NF54 68.2% 40.1% **----TGAATGTGTTGC----------------------------------------------------------------**  6 CAMP/Malaysia 62.8% 38.2% **----TGAATGTGTTGC----------------------------------------------------------------**  7 HB3 86.6% 36.4% **CTATCAGAAGTATTTT---GTGGATGATTATTTCTAGAACCATGCAT-------------ATGTCCATGTTGTCCTGTAC**  consensus/100% **................................................................................**  consensus/90% **................................................................................**  consensus/80% **................................................................................**  consensus/70% **....suussGTusTss................................................................**  cov pid **321**  **. ]** **338**  1 FCB2_msp2 100.0% 100.0% **GGTCATACTTC-------**  2 3D7 63.6% 65.9% **------------------**  3 7G8 48.5% 88.3% **------------------**  4 FVO 74.9% 33.7% **TTTAATTACCT---TCAT**  5 NF54 68.2% 40.1% **------------------**  6 CAMP/Malaysia 62.8% 38.2% **------------------**  7 HB3 86.6% 36.4% **CTTTATTCTCTGGTGCAG**  consensus/100% **..................**  consensus/90% **..................**  consensus/80% **..................**  consensus/70% **..................** |
